# Comparing the Impact of Different Antiarrhythmic Classes on Clinical Outcomes Following Atrial Fibrillation Catheter Ablation

**DOI:** 10.3390/ph18071022

**Published:** 2025-07-10

**Authors:** Andrej Belančić, Yusuf Ziya Sener, Metin Oksul, Cansu Ozturk, Serdar Soner, Adnan Duha Comert, Gamze Yeter Arslan, Dinko Vitezić, Bojan Jelaković, Erkan Baysal

**Affiliations:** 1 Department of Basic and Clinical Pharmacology with Toxicology, Faculty of Medicine, University of Rijeka, Braće Branchetta 20, 51000 Rijeka, Croatia; dinko.vitezic@uniri.hr; 2 Department of Cardiology, Thoraxcenter, Erasmus University Medical Center, 51000 Rotterdam, The Netherlands; yzsener@yahoo.com.tr; 3 Department of Cardiology, Gazi Yasargil Training and Research Hospital, 21010 Diyarbakir, Türkiye; moksul_73@hormail.com (M.O.); cnsozt@yahoo.com (C.O.); drserdar_89@hotmail.com (S.S.); drtrapezus@gmail.com (A.D.C.); dr.erkan.baysal@hotmail.com (E.B.); 4 Department of Cardiology, Kepez State Hospital, 07320 Antalya, Türkiye; gaslan@kuh.ku.edu.tr; 5 Department of Nephrology, Arterial Hypertension, Dialysis and Transplantation, University Hospital Center Zagreb, 10000 Zagreb, Croatia; jelakovicbojan@gmail.com

**Keywords:** antiarrhythmics, amiodarone, atrial fibrillation, catheter ablation, flecainide, propafenone

## Abstract

**Background/Objectives**: Catheter ablation has become the standard of care for patients with symptomatic and drug-refractory atrial fibrillation (AF). Both Class IC and Class III antiarrhythmic drugs (AADs) are effective in preventing early recurrences of AF, but not late recurrences, compared with the usual care. We aimed to compare the effects of two months of Class IC versus Class III AADs following AF catheter ablation on clinical outcomes, including arrhythmia recurrence and safety endpoints. **Methods**: All patients undergoing AF catheter ablation between January 2015 and November 2024 were screened, and cases meeting the inclusion criteria were included. Primary outcome was defined as atrial tachycardia recurrence-free survival. **Results**: A total of 98 patients (mean age 54.2 ± 14.0 years; 55.1% male) were enrolled, with 66.3% presenting with paroxysmal atrial fibrillation (AF). The mean left atrial diameter was 38.7 ± 5.1 mm, and 78.6% underwent cryoballoon ablation. Class IC AADs were administered to 62 cases, while the remaining 36 patients received amiodarone following catheter ablation. The rate of atrial tachycardia (ATa) recurrence was comparable between the patients treated with Class IC and Class III AADs (9.7% vs. 19.4%; *p* = 0.169). Predictors of ATa recurrence were identified as history of direct current cardioversion—DCCV (HR: 5.86; 95%CI: 1.44–23.82)—and LA diameter (HR: 1.17; 95%CI: 1.04–1.31). The most frequent AAD-related adverse event was symptomatic bradycardia (6.1%), which resolved in all cases following dose reduction. **Conclusions**: Class IC and Class III antiarrhythmics show comparable efficacy in terms of preventing ATa recurrence following AF catheter ablation. AAD-related adverse event rates are negligible for short-term use.

## 1. Introduction

Atrial fibrillation (AF) is the most common sustained cardiac arrhythmia, significantly contributing to morbidity, mortality, and healthcare burden [1]. Catheter ablation has emerged as a cornerstone therapy for rhythm control, particularly in symptomatic and drug-refractory AF patients [2,3]. However, post-ablation arrhythmia recurrence remains a critical challenge, necessitating the adjunctive use of antiarrhythmic drugs (AADs) to maintain sinus rhythm during the vulnerable post-procedural period [4].

The optimal choice of AADs following AF ablation remains a subject of debate. Class IC and III antiarrhythmic drugs block sodium and potassium channels and prolong the action potential duration of atrial myocytes. Both Class IC and Class III AADs are used to improve the heterogeneity of atrial action potential duration [5]. While amiodarone (Class III AAD) is renowned for its potent antiarrhythmic properties and efficacy in maintaining sinus rhythm, its long-term use is limited by systemic toxicities affecting thyroid, pulmonary, and hepatic function. On the other hand, Class IC AADs, such as flecainide and propafenone, offer a favorable side effect profile but may be less effective. Moreover, Class IC AADs exert proarrhythmic effects, and they are established to be associated with increased mortality risk in patients with structural heart diseases in previous trials [6,7]. A few studies have evaluated the efficacy of Class IC AADs and amiodarone compared to the usual care in preventing the recurrence of atrial arrhythmias following catheter ablation. These studies have shown that AADs are beneficial in mitigating early recurrences but do not show clear benefit in terms of long-term clinical outcomes and have considerable side effects [8,9,10]. Although existing data suggests the potential benefits of more effective short-term suppression of atrial arrhythmia recurrence, the data is not strong enough to take precedence over direct evidence [11]. There are no studies comparing the impact of different AAD classes following catheter ablation with regard to preventing arrhythmia recurrences and the safety profile. The lack of direct comparative data between AAD classes in the post-ablation setting leaves clinicians with limited guidance on individualized treatment strategies [6,12].

Given the growing emphasis on personalized medicine in electrophysiology, determining the most effective and safest AAD strategy for post-ablation is crucial. While some studies suggest amiodarone’s superior efficacy in preventing arrhythmia recurrence following conversion to sinus rhythm, its safety concerns in long-term use make Class IC AADs an attractive alternative for suitable patients [13]. Additionally, patient-specific factors—such as age, comorbidities, including heart failure, hypertension, and coronary disease, and the presence of left ventricular hypertrophy—may influence drug selection and treatment response [14]. Understanding how these variables interact with different AAD regimens can refine clinical decision-making, ensuring that therapy is tailored to maximize benefit while minimizing risk.

Although short-term post-ablation AAD therapy reduces early recurrences and arrhythmia-related hospitalizations, AAD selection is recommended to be individualized based on patient risk profiles [15]. However, no head-to-head trial has evaluated the short-term use of Class IC versus Class III antiarrhythmic drugs in terms of atrial tachycardia recurrence and safety outcomes following atrial fibrillation catheter ablation. This study addresses this literature gap by evaluating the impact of two months of Class IC versus Class III AAD therapy following AF catheter ablation on clinical outcomes. By leveraging real-world clinical data, we aim to provide insights into the optimal post-ablation antiarrhythmic treatment strategy, guiding individualized therapy selection and improving patient outcomes.

## 2. Results

### 2.1. Baseline Characteristics

A total of 98 patients were enrolled in the study. The mean age of the study population was 54.2 ± 14.0 years, and 55.1% of the cases (*n* = 54) were male. Hypertension was present in 69 (70.4%), diabetes in 22 (22.4%), coronary artery disease in 52 (53.1%), and ischemic stroke in 9 (9.2%) patients. The mean value of left atrial (LA) diameter was 38.7 ± 5.1 mm, and the mean left ventricular ejection fraction (LVEF) value was 57.6 ± 5.4%. The majority of patients (66.3%) had paroxysmal AF, with a median CHADS-VA score of 1 (0–6) and a median HAS-BLED score of 1 (0–4). Class IC antiarrhythmic drugs were administered in 62 (63.3%) patients, while amiodarone was administered to the remaining 36 (36.7%) cases at least 2 months following catheter ablation. Patients treated with Class IC AAD were found to be younger (51.1 ± 15.3 vs. 59.5 ± 9.7; *p* = 0.001). Comorbidities—including hypertension, diabetes, and coronary artery disease—were more common in the Class III group, which also had a higher median CHADS-VA score and larger LA diameter compared with the Class IC group (Table 1).

### 2.2. Procedural Characteristics

The baseline rhythm was sinus rhythm in the majority of patients (74.5%), with cryoballoon ablation being performed in the majority of cases (78.6%) and pulmonary vein isolation alone being performed in 87.8% of cases. The ablation procedure was successful in 96 (98%) cases. The median follow-up period for the recurrence of atrial tachyarrhythmias was 17.5 (1.5–120.0) months, during which 13 (13.3%) patients experienced recurrence of atrial tachycardia. The ablation procedure-related characteristics were found to be similar between patients treated with Class IC and Class III AAD following catheter ablation (Table 2, Supplementary Figure S1). Procedural characteristics are illustrated in Supplementary Figure S1.

### 2.3. Atrial Tachycardia Recurrence

**Figure 1:**
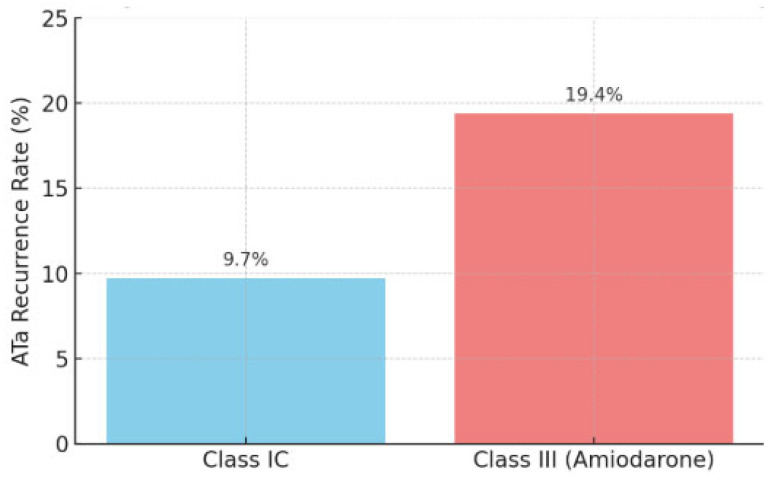
The figure illustrates the bar graph of atrial tachycardia recurrence rates following atrial fibrillation catheter ablation.

Atrial tachycardia recurrence was observed in 19.4% of patients in the Class III group (seven patients) and 9.7% in the Class IC group (six patients), with no statistically significant difference (*p* = 0.169) (Figure 1).

**Figure 2:**
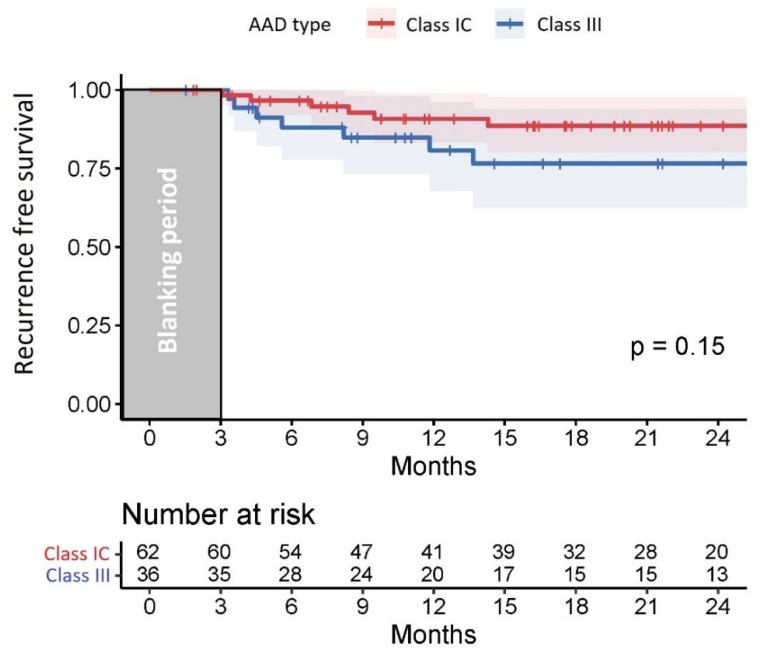
Kaplan–Meier curve shows the comparable atrial tachycardia-free survival rates among Class IC and Class III group.

No statistically significant difference in recurrence-free survival was observed between the patients treated with Class III and those treated with Class IC AAD (Figure 2).

Persistent AF (HR: 5.38; 95%CI: 1.65–17.50), a history of DCCV (HR: 5.41; 95%CI: 1.66–17.62), duration of AF (HR: 1.13; 95%CI: 1.06–1.20), baseline AF rhythm (HR: 3.99; 95%CI: 1.34–11.9), and LA diameter (HR: 1.14; 95%CI: 1.03–1.27) were found to be associated with atrial tachycardia recurrence in univariable Cox regression analysis. Subsequent multivariable Cox regression analysis revealed that only history of DCCV (HR: 5.86; 95%CI: 1.44–23.82) and LA diameter (HR: 1.17; 95%CI: 1.04–1.31) were the independent predictors of atrial tachycardia recurrence (Table 3).

### 2.4. Safety Endpoints

Symptomatic bradycardia occurred in six cases (6.1%), but all cases became asymptomatic with modification of the drug’s dose. Symptomatic bradycardia occurred within the first two weeks in all cases, with a propafenone dose of 150 mg twice daily for three patients undergoing treatment with Class IC AAD, and 200 mg twice daily for the remaining three patients undergoing treatment with amiodarone. None of the patients experienced syncope, and their complaints were lightheadedness and presyncope. All patients recovered following a reduction in dosage, and drug discontinuation was not needed in any cases. Dyspnea developed in three patients who were treated with propafenone, but patients could tolerate it in time, and drug discontinuation was not needed in any cases. In one patient treated with amiodarone, transient ischemic attack occurred one week after the catheter ablation, and AF recurrence was not documented in that case. Increased liver transaminases were observed in only one patient treated with amiodarone at the conclusion of a two-month treatment period, and these returned to normal levels following drug discontinuation. One patient treated with amiodarone was diagnosed with hypothyroidism 40 days after catheter ablation and underwent a short course of levothyroxine replacement following the completion of amiodarone treatment. The incidence of safety endpoints was comparable across the study groups (Table 4).

## 3. Discussion

Our study demonstrated that the type of AAD used following AF catheter ablation did not significantly impact atrial tachycardia recurrence-free survival. While both Class IC AAD and Class III AAD were effective in maintaining sinus rhythm, baseline differences between the treatment groups highlight important considerations for post-ablation management. Patients treated with amiodarone were older and had a higher burden of comorbidities, including hypertension, diabetes, and coronary artery disease, as well as larger left atrial diameters and higher CHADS-VA scores. These factors likely contributed to the observed higher trends in atrial tachycardia recurrence in the Class III AAD arm. Multivariable analysis identified a history of direct current cardioversion (DCCV) and LA diameter as independent predictors of recurrence, aligning with prior studies emphasizing structural remodeling and electrical vulnerability as key determinants of post-ablation outcomes. Importantly, safety endpoints—including symptomatic bradycardia, dyspnea, and thyroid or hepatic dysfunction—were rare and comparable between groups. These findings suggest that while both drug classes are viable options for rhythm control, patient characteristics should inform individualized treatment decisions to optimize outcomes.

The administration of AADs after AF catheter ablation was shown to reduce readmission rates within 90 days, and amiodarone was associated with the greatest reduction in readmission rates, while dronaderone and Class IC agents had no significant effect [16]. Several studies established the benefit of short-term (≤3 months) use of AADs after catheter ablation on early (within the first 3 months) atrial tachycardia recurrence, while it had no effect on late recurrence rates [5,10,17]. However, continued use of AADs beyond 3 months was associated with a reduced atrial tachycardia recurrence rate at 1 year following AF ablation [18]. In addition to the lack of benefit with regard to late atrial tachycardia recurrences, use of AADs was linked to reduced treatment satisfaction at the 1-year follow-up of AF ablation in patients with paroxysmal AF [19]. The existing literature includes comparisons of the use of AADs versus usual care, and our findings reveal the first results of comparing the impact of different AAD classes on late (beyond 3 months after AF ablation) atrial tachycardia recurrence rates after AF catheter ablation. We found that atrial tachycardia recurrence-free survival was comparable between short-term administration of Class IC and Class III drugs. The comparable atrial tachycardia-free survival observed between the two groups likely reflects the similar short-term efficacy of both AAD classes in rhythm control following ablation. This similarity may also be attributable to the exclusion of patients with structural heart disease, thereby reducing clinical heterogeneity. Furthermore, the brief duration of therapy—particularly the limited two-month use of amiodarone—may have attenuated potential differences in efficacy. However, our findings highlight the predominance of safety considerations over efficacy in guiding the selection of the short-term AAD class following AF catheter ablation in patients without structural heart disease.

There are several defined predictors of atrial tachycardia recurrence after AF catheter ablation, and many scores, including the BASE-AF2, ALARMEc, and APPLE scores, were developed to estimate the risk of recurrence [20]. Predictors of early recurrence (within 3 months) were defined as hypertension, LA enlargement, lack of vena cava isolation and permanent AF, and termination of AF during ablation [21,22]. Obesity, presence of early recurrence, right and left atrial enlargement, AF duration, and more than two procedural attempts were defined as the predictors of late recurrence [23,24,25]. The history of DCCV and increased LA diameter were found to be predictors of atrial tachycardia recurrence in our study. The relationship between LA diameter and recurrence demonstrates the importance of structural remodeling, and the association between previous DCCV and recurrence indicates the importance of both structural and electrical remodeling in the pathogenesis of recurrence.

Antiarrhythmic medications have been proven to be effective in the prevention of recurrence of atrial tachycardia after AF ablation; however, they can also be associated with certain adverse events. Amiodarone, for example, has been shown to cause thyroid dysfunction, elevated liver transaminases, and pulmonary and corneal toxicity [26,27]. Similarly, propafenone has been associated with dyspnea [28]. Furthermore, both Class IC and III AADs have been associated with QTc prolongation and bradycardia [25]. The most common AAD-related side effect was symptomatic bradycardia, with an incidence of 6.1% observed in our study. Sick sinus syndrome has been reported following the termination of long-term persistent tachycardia due to the inhibition of the sinus node [29], potentially contributing to AAD-related bradycardia following catheter ablation. The prevalence of adverse effects such as dyspnea, liver dysfunction, and thyroid dysfunction was found to be negligible. This finding indicates that the safety profile of the short-term use of AADs following AF catheter ablation is acceptable.

The selection of an AAD for patients with AF requires careful consideration, as AADs can have several side effects. Several variables, including age, underlying comorbidities, the presence of structural heart disease, baseline heart rate, and frailty, should be taken into consideration before prescribing an AAD [30,31]. The latest ESC guideline for the management of atrial fibrillation underlines the importance and priority of safety rather than the efficacy in selecting the AAD [32]. Due to the retrospective nature of our study, there were differences in baseline characteristics across the treatment arms (younger patients with fewer comorbidities in the Class IC arm, compared to older patients with more comorbidities in the amiodarone arm). We were unable to perform a regression analysis to identify predictors of adverse events; this resulted in a lack of data to inform suggestions regarding AAD selection criteria. Our findings showed the similar efficacy and safety profile of Class IC and Class III antiarrhythmic drugs in short-term use after AF catheter ablation in patients without structural heart disease and normal left ventricular ejection fraction. However, these findings cannot be generalized to the entire population, and general recommendations should be applied to long-term use and patients with comorbidities.

### Strengths and Limitations

A key strength of this study is its real-world applicability, as it includes a well-characterized cohort of post-ablation atrial fibrillation patients with diverse clinical profiles. The study benefits from comprehensive baseline assessments and detailed procedural data, allowing robust comparison of the efficacy and safety of Class IC AAD versus amiodarone. The inclusion of long-term follow-up data further strengthens our findings, particularly in identifying predictors of atrial tachyarrhythmia recurrence. Additionally, the study’s rigorous statistical approach, including multivariable analysis, enhances the validity of the reported associations.

However, several limitations should be acknowledged. The study is observational, making it susceptible to residual confounding despite statistical adjustments. The relatively small sample size, particularly in the Class III (amiodarone) group, may limit the generalizability of our findings. The study did not assess long-term side effects of amiodarone beyond the follow-up period, which is a key consideration given its known toxicity profile. Therefore, although AAD is generally used in the short-term following AF catheter ablation, these results cannot be generalized to patients who have used AAD in the long-term after catheter ablation. Finally, the recurrence of atrial tachycardia was assessed through retrospective records, and continuous monitoring was not performed during follow-up in most cases. The absence of recurrence screening based on continuous monitoring might have led to under-detection of recurrences and detection bias. Future randomized studies with larger cohorts and extended follow-up are needed to validate these findings.

## 4. Materials and Methods

### 4.1. Study Population

**Figure 3:**
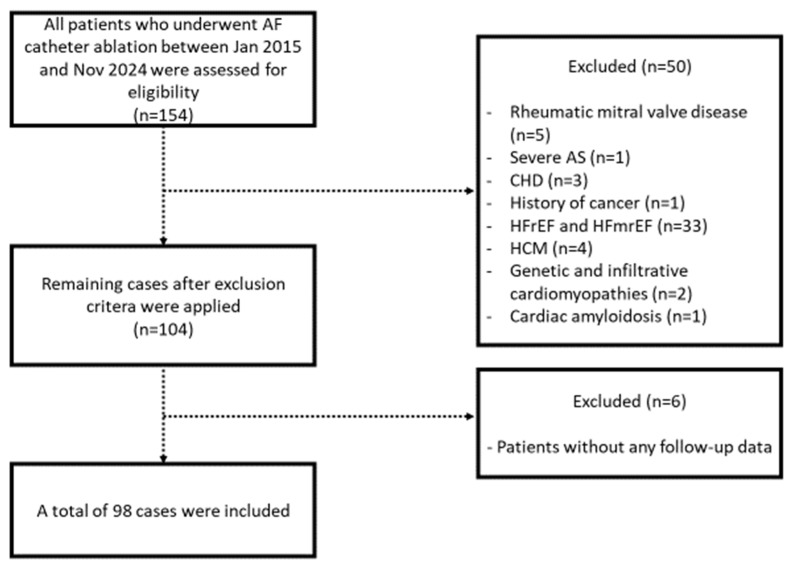
Flow diagram for the study population. AF, atrial fibrillation; AS, aortic stenosis; CHD, congenital heart disease; HFrEF, heart failure with reduced ejection fraction; HFmrEF, heart failure with mildly reduced ejection fraction; HCM, hypertrophic cardiomyopathy.

In this retrospective observational study, all patients who underwent AF catheter ablation at Gazi Yasargil Training and Research Hospital between January 2015 and November 2024 were screened. Patients aged 18 years and older with available periprocedural and follow-up data meeting inclusion criteria were included. Patients with underlying comorbidities, including severe aortic stenosis, congenital heart disease, rheumatic mitral valve disease, heart failure with reduced and mildly reduced ejection fraction (left ventricular ejection fraction < 50%), cardiac amyloidosis, and hypertrophic, genetic, and infiltrative cardiomyopathies, were excluded. Moreover, patients with a history of cancer and a significant coronary artery stenosis without revascularization were also excluded (Figure 3). The study was approved by Gazi Yasargil Training and Research Hospital Ethics Committee (7 February 2025; no: 350).

### 4.2. Pre-Procedural Management

All patients underwent transthoracic echocardiography to evaluate LA diameter and LVEF before the procedure. Transesophageal echocardiography was performed to exclude LA and LA appendage thrombus. The procedure was performed without discontinuation of anticoagulant drugs, and antiarrhythmic agents were discontinued 5 half-times before the procedure (except amiodarone).

### 4.3. Cryoablation Procedure

In patients undergoing cryoablation, only pulmonary vein isolation was aimed at during the index procedure, and no additional ablation was planned. The procedures were performed under conscious anesthesia. A femoral venous and arterial route (for pigtail catheter) was used for catheter placement, and transseptal puncture was performed for access to the LA. The procedure was started with a bolus of heparin as an anticoagulant, and maintenance therapy was performed according to ACT monitoring. The ablation procedure was performed with a 28 mm CB-2 (Arctic Front Advance; Medtronic, Inc., Maharashtra, India) cryoballoon placed in the antral region of the pulmonary veins using the over-the-wire technique. The CB-2 balloon was advanced over a J-tip guidewire and a specialized mapping catheter (Achieve Mapping Catheter; Medtronic, Inc.). CB-2 catheters were frozen in the antral region of the pulmonary veins in accordance with previous clinical studies, and acute pulmonary vein isolation was confirmed by demonstrating exit and entrance block.

### 4.4. Radiofrequency (RF) Ablation Procedure

RF ablation was performed under deep sedation or general anesthesia. After transseptal puncture, a steerable sheath (Agilis, St Jude Medical, St Paul, MN, USA) was advanced into the left atrium under fluoroscopic guidance. After heparin bolus, additional heparin doses were administered so that the ACT level was 300–350 s. A 3-dimensional electroanatomic mapping system (EnSite Precision, Abbott (Chicago, IL, USA) or CARTO, Biosense Webster (Irvine, CA, USA)) was used to map the left atrium with a multipolar catheter (Advisor HD Grid, Abbott, or Lasso, Pentaray, Biosense Webster). The pulmonary veins were then isolated with an irrigated catheter (TactiCath, Abbott or SmartTouch, Biosense Webster). Large antral pulmonary vein isolation was performed in all cases. Non-pulmonary vein ablation was decided according to operator preference and clinical condition of the patient. Dissociation of pulmonary vein signals and entrance/exit blocks were accepted as criteria for acute procedural success.

### 4.5. Post-Procedural Management

Transthoracic echocardiography was performed to assess post-procedural complications. Patients without bleeding complications received their first oral anticoagulant dose 2–3 h after sheath removal. Patients without complications were discharged one day after the procedure. Antiarrhythmic therapy was continued for 2 months in patients without contraindications or disabling side effects. The choice of antiarrhythmic drug was based on the clinician’s experience and preference. Propafenone and flecainide were the Class IC AADs, while amiodarone was the only Class III AAD used. All patients were treated with oral anticoagulation (OAC) for the first 2 months, regardless of the risk of embolism. After 2 months, OAC treatment was decided according to the patient’s CHADS-VA score. Patients with high scores were treated with OAC indefinitely. Follow-up visits were scheduled for 1, 3, 6, and 12 months.

### 4.6. Outcomes

An atrial arrhythmia episode lasting longer than 30 s was considered as atrial tachycardia recurrence [33]. Atrial arrhythmia recurrences were detected using 12-lead electrocardiography (ECG) and 24 h Holter monitoring. A 12-lead ECG was performed during every follow-up visit for all patients. At the clinician’s discretion, 24 h Holter rhythm monitoring was performed on symptomatic patients with a normal ECG and on some asymptomatic patients. The first 3 months after the procedure were considered a blanking period, and atrial arrhythmias during the first 3 months were not accepted as recurrences. Atrial tachycardia-free survival was considered as the primary endpoint. Safety endpoints were determined as the occurrence of transient ischemic attack or ischemic stroke and drug-related adverse events, including dyspnea, symptomatic bradycardia, thyroid dysfunction, and increased liver transaminases.

### 4.7. Statistical Analysis

Normality of distribution was tested using the Kolmogorov–Smirnov test. Normally distributed continuous variables were presented as mean ± standard deviation, while non-parametric variables were presented as median (min–max). Categorical variables were expressed as frequencies and percentages. The student’s t-test was used for parametric variables and the Mann–Whitney U test for non-parametric variables when comparing independent groups. The χ^2^ test or Fisher’s exact test, as appropriate, was used to compare percentages of categorical variables. Univariable Cox proportional hazards were estimated for parameters predicting atrial tachycardia recurrence, and *p*-values < 0.200 were further included in multivariable Cox regression analysis. Kaplan–Meier analysis with the log-rank test was used to compare atrial tachycardia-free survival. *p* values < 0.05 were considered statistically significant. R (version 4.1.2) software was used for analysis.

## 5. Conclusions

In conclusion, our findings indicate that Class IC antiarrhythmic drugs and amiodarone exhibit comparable efficacy in maintaining sinus rhythm following AF catheter ablation, with no significant differences in safety profiles. Despite the limited sample size, these results support the clinical use of both AAD classes without concerns regarding efficacy, suggesting that selection should be guided primarily by patients’ underlying comorbidities. By addressing a critical gap in rhythm management, this study provides valuable evidence to guide clinical decision-making and improve long-term outcomes in patients undergoing AF catheter ablation. Larger multicenter randomized trials are warranted to validate these findings.

## Figures and Tables

**Table 1:** Baseline characteristics of the study population.

	All Patients *n* = 98	Class IC AAD *n* = 62	Class III AAD *n* = 36	*p*-Value
Age, years	54.2 ± 14.0	51.1 ± 15.3	59.5 ± 9.7	0.001 **
Male sex, *n* (%)	54 (55.1)	37 (59.7)	17 (47.2)	0.232
AF type, paroxysmal	65 (66.3)	43 (69.4)	22 (61.1)	0.405
Prior DCCV	34 (34.7)	17 (24.4)	17 (47.2)	0.047 *
History of AF ablation	11 (11.2)	6 (9.7)	5 (13.9)	0.524
Duration of AF (months)	8 (1–36)	7 (2–36)	8 (1–24)	0.703
CHADS-VA	1 (0–6)	1 (0–6)	2 (0–5)	0.004 **
HAS-BLED	1 (0–4)	1 (0–4)	1 (0–3)	0.012 *
**Comorbidities**				
Hypertension	69 (70.4)	39 (62.9)	30 (83.3)	0.033 *
Diabetes mellitus	22 (22.4)	9 (14.5)	13 (36.1)	0.014 *
Coronary artery disease	52 (53.1)	26 (41.9)	26 (72.2)	0.004 **
Ischemic stroke	9 (9.2)	3 (4.8)	6 (16.7)	0.071
**Medications, *n* (%)**				
Beta blockers	77 (78.6)	47 (75.8)	30 (83.3)	0.381
RAAS inhibitors	53 (54.1)	26 (41.9)	27 (75.0)	0.002 **
Digoxin	13 (13.3)	8 (12.9)	5 (13.9)	0.890
Anticoagulants	87 (88.8)	54 (87.1)	33 (91.7)	0.490
Antiarrhythmic drugs	64 (65.3)	41 (66.1)	23 (63.9)	0.822
**Echocardiographic parameters**				
LA diameter, mm	38.7 ± 5.1	37.7 ± 4.2	40.5 ± 6.0	0.026 *
LVEF, %	57.6 ± 5.4	58.0 ± 5.4	56.8 ± 5.4	0.336
Moderate and severe MR, *n* (%)	21 (23.3)	10 (17.9)	11 (32.4)	0.115
**Laboratory values**				
Hemoglobin, g/dL	14.0 ± 1.9	14.1 ± 1.8	13.8 ± 2.0	0.396
GFR, mL/min/m^2^	90 (23–125)	90 (23–125)	90 (54–110)	0.468

**Abbreviations**: AF: Atrial fibrillation, AAD: Antiarrhythmic drugs, DCCV: Direct current cardioversion, RAAS: Renin angiotensin aldosterone system, LA: Left atrial, LVEF: Left ventricular ejection fraction, MR: Mitral regurgitation, GFR: Glomerular filtration rate. * Statistically significant *p*-values < 0.05. ** Statistically significant *p*-values < 0.01.

**Table 2:** Procedural characteristics of the patients.

	All Patients *n* = 98	Class IC AAD *n* = 62	Class III AAD *n* = 36	*p*-Value
Baseline rhythm, *n* (%)				0.067
- Sinus	73 (74.5)	50 (80.6)	23 (63.9)	
- AF	25 (25.5)	12 (19.4)	13 (36.1)	
Ablation technique, *n* (%)				0.243
- Cryoablation	77 (78.6)	51 (82.3)	26 (72.2)	
- RF ablation	21 (21.4)	11 (17.7)	10 (27.8)	
Ablation type, *n* (%)				0.309
- PVI only	86 (87.8)	56 (90.3)	630 (83.3)	
- PVI plus	12 (12.2)	6 (9.7)	6 (16.7)	
Common ostium, *n* (%)	2 (2)	2 (3.2)	0 (0.0)	2.530
Procedural success, *n* (%)	96 (98)	61 (98.4)	35 (97.2)	1.000
ATa recurrence, *n* (%)	13 (13.3)	6 (9.7)	7 (19.4)	0.169
Follow-up (months)	17.5 (1.5–120.0)	19.1 (1.8–120.0)	14.1 (1.5–84.0)	0.446

**Abbreviations**: AAD: Antiarrhythmic drug, AF: Atrial fibrillation, RF: Radiofrequency, ATa: Atrial tachyarrhythmia, PVI: Pulmonary vein isolation, PVI plus: Pulmonary vein isolation with additional ablation.

**Table 3:** Predictors of atrial tachycardia recurrence.

Variables	Univariable HR (95%CI)	*p*-Value	Multivariable HR (95%CI)	*p*-Value
Age, years	1.02 (0.98–1.06)	0.222		
Male sex	0.53 (0.17–1.63)	0.270		
Hypertension	0.92 (0.28–3.00)	0.925		
Diabetes mellitus	1.46 (0.45–4.76)	0.526		
Coronary artery disease	0.77 (0.26–2.30)	0.645		
Type of AF				
Persistent vs. Paroxysmal	5.38 (1.65–17.50)	0.005 *	1.76 (0.32–9.76)	0.513
Prior DCCV	5.41 (1.66–17.62)	0.005 *	5.86 (1.44–23.82)	0.013 *
AF duration (months)	1.13 (1.06–1.20)	<0.001 *	1.07 (0.99–1.16)	0.082
PVI plus vs. PVI only	0.85 (0.11–6.57)	0.879		
Baseline rhythm				
AF vs. Sinus rhythm	3.99 (1.34–11.9)	0.013 *	2.06 (0.40–10.69)	0.386
Ablation type				
RF vs. Cryoablation	0.88 (0.19–4.06)	0.889		
LA diameter, mm	1.14 (1.03–1.27)	0.008 *	1.17 (1.04–1.31)	0.008 **
LVEF, %	1.01 (0.91–1.11)	0.829		
AAD at discharge				
Class III vs. Class IC AAD	2.17 (0.73–6.48)	**0.162**	1.57 (0.43–5.64)	0.489

**Abbreviations**: AAD: Antiarrhythmic drug, AF: Atrial fibrillation, DCCV: Direct current cardioversion, LA: Left atrium, LVEF: Left ventricular ejection fraction, RF: Radiofrequency, PVI: Pulmonary vein isolation, PVI plus: Pulmonary vein isolation with additional ablation. * Statistically significant *p*-values < 0.05. ** Statistically significant *p*-values < 0.01.

**Table 4:** Safety endpoints in patients treated with class IC and class III AAD.

	All Patients *n* = 98	Class IC AAD *n* = 62	Class III AAD *n* = 36	*p*-Value
Symptomatic bradycardia (<60 bpm)	6 (6.1)	3 (4.8)	3 (8.3)	0.487
Dyspnea	3 (3.1)	3 (4.8)	0 (0.0)	0.296
Stroke/TIA	1 (1)	0 (0.0)	1 (2.8)	0.367
Increase in liver transaminases (x3 ULN)	1 (1)	0 (0.0)	1 (2.8)	0.367
Thyroid dysfunction	1 (1)	0 (0.0)	1 (2.8)	0.367

**Abbreviations**: AAD: Antiarrhythmic drug, TIA: Transient ischemic attack, ULN: Upper limit of normal.

## Data Availability

The original contributions presented in this study are included in the article, further inquiries can be directed to the corresponding author.

## Supplementary Materials

The following supporting information can be downloaded at: https://www.mdpi.com/article/10.3390/ph18071022/s1, Figure S1: Baseline and procedural characteristics by group.
