# Supplementary figures and images for: Comparing the Impact of Different Antiarrhythmic Classes on Clinical Outcomes Following Atrial Fibrillation Catheter Ablation
Source: Pharmaceuticals (Basel). 2025 Jul 10;18(7):1022. doi: 10.3390/ph18071022 (PMC12299593; doi:10.3390/ph18071022)

## Supplementary Materials

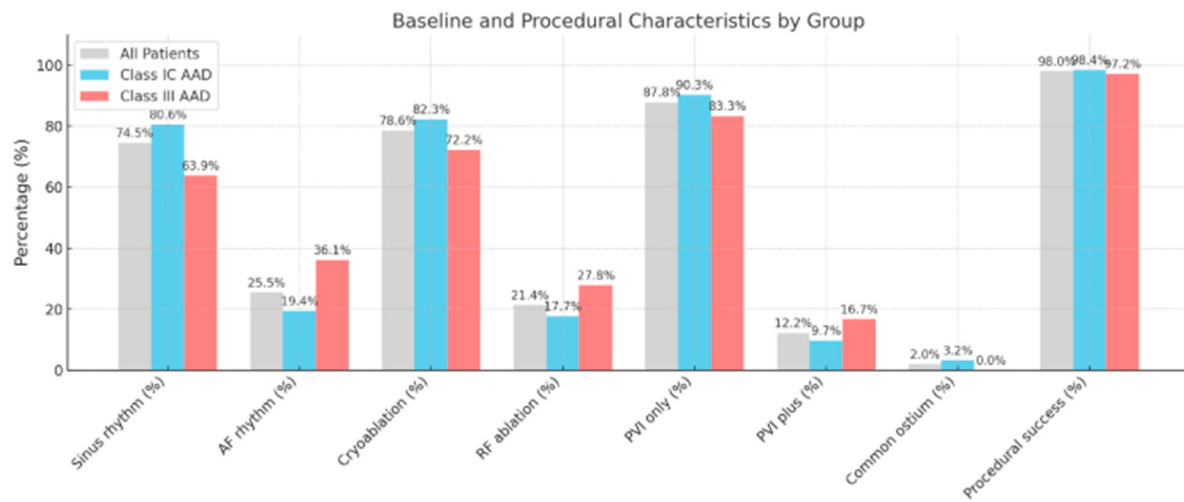

**Figure S1.** Baseline and procedural characteristics by group.

Supplement: Supplementary file 1 [file pharmaceuticals-18-01022-s001.zip › pharmaceuticals-3715200-supplementary.pdf]
